# “*A false sense of confidence*” The perceived role of inflammatory point-of-care testing in managing urinary tract infections in Dutch nursing homes: a qualitative study

**DOI:** 10.1186/s12877-020-01853-9

**Published:** 2020-11-04

**Authors:** S. D. Kuil, C. Schneeberger, F. van Leth, M. D. de Jong, J. Harting

**Affiliations:** 1 Department of Medical Microbiology, Amsterdam Infection & Immunity Institute, Amsterdam UMC, University of Amsterdam, Meibergdreef 9, Amsterdam, The Netherlands; 2 Amsterdam Institute for Global Health and Development, Amsterdam, The Netherlands; 3 Department of Global Health, Amsterdam UMC, University of Amsterdam, Meibergdreef 9, Amsterdam, The Netherlands; 4 Department of Public and Occupational Health, Amsterdam Public Health Research Institute, Amsterdam UMC, University of Amsterdam, Meibergdreef 9, Amsterdam, The Netherlands

**Keywords:** Nursing home, Urinary tract infection, Point-of-care test, Implementation, Qualitative study

## Abstract

**Background:**

Diagnosing urinary tract infections (UTI) in nursing home residents is complex, due to frequent non-specific symptomatology and asymptomatic bacteriuria. The objective of this study was to explore health care professionals’ perceptions of the proposed use of inflammatory marker Point-Of-Care Testing (POCT) in this respect.

**Methods:**

We conducted a qualitative inquiry (2018–2019) alongside the multicenter PROGRESS study (NL6293), which assessed the sensitivity of C-reactive protein and procalcitonin POCT in UTI. We used semi-structured face-to-face interviews. The participants were physicians (*n* = 12) and nurses (*n* = 6) from 13 nursing homes in the Netherlands. Most respondents were not familiar with inflammatory marker POCT, while some used POCT for respiratory tract infections. Both the interview guide and the analysis of the interview transcripts were based on the Consolidated Framework for Implementation Research.

**Results:**

All respondents acknowledged that sufficiently sensitive POCT could decrease diagnostic uncertainty to some extent in residents presenting with non-specific symptoms. They primarily thought that negative test results would rule out UTI and justify withholding antibiotic treatment. Secondly, they described how positive test results could rule in UTI and justify antimicrobial treatment. However, most respondents also expected new diagnostic uncertainties to arise. Firstly, in case of negative test results, they were not sure how to deal with residents’ persisting non-specific symptoms. Secondly, in case of positive test results, they feared overlooking infections other than UTI. These new uncertainties could lead to inappropriate antibiotics use. Therefore, POCT was thought to create a false sense of confidence.

**Conclusions:**

Our study suggests that inflammatory marker POCT will only improve UTI management in nursing homes to some extent. To realize the expected added value, any implementation of POCT requires thorough guidance to ensure appropriate use. Developing UTI markers with high negative and positive predictive values may offer greater potential to improve UTI management in nursing homes.

**Supplementary Information:**

The online version contains supplementary material available at 10.1186/s12877-020-01853-9.

## Background

The most common infections in nursing home residents involve the urinary tract [1–3]. At any age, the diagnosis of urinary tract infection (UTI) is mainly based on the presence of urinary symptoms (urgency, frequency, dysuria and supra-pubic tenderness) [4, 5], but this poses difficulties in the case of nursing home residents. Firstly, the ability to express specific urinary tract symptoms is often limited if residents suffer from cognitive impairments [6, 7]. Secondly, the only presenting feature of UTI may be non-specific symptoms [8]. Such symptoms are frequently reported in nursing home residents, but could be attributed to many causes, including deterioration of underlying cognitive impairments [9]. Finally, in nursing home residents there is a high prevalence of asymptomatic bacteriuria (ASB), i.e. the presence of bacteria in the urine, without clinical signs of infection [10–12]. Therefore, urine tests to detect bacteria, such as dipstick analysis and bacterial culture, lack specificity in diagnosing UTI. For this reason, most guidelines on UTI in nursing home residents do not recommend the use of dipstick urinalysis, or only recommend it to ‘rule out’ UTI [13, 14]. Due to these difficulties in recognizing and diagnosing UTI, inappropriate antibiotic use is common, which consequently increases the risk of antimicrobial resistance development [15, 16].

Diagnostic tests to confirm UTI in nursing home residents are not yet available. Our current research focuses on the sensitivity of two blood inflammatory markers (C-reactive protein [CRP] and procalcitonin [PCT]), measured in point-of-care testing (POCT) to support diagnosing UTI [17]. Provided the sensitivity of such POCT is sufficient, it can be used in the clinical work-up to confirm the UTI diagnosis in nursing home residents, provided that the positive predictive value is sufficient in that population. This would enable residents with true UTI to be identified, potentially improving the appropriate use of antibiotics, and consequently reducing unnecessary side-effects and antimicrobial resistance. As CRP and PCT are general inflammatory markers, levels might also increase in response to various other inflammatory or infectious diseases. It is therefore important to use POCT in UTI as a complement to the current clinical work-up by the attending physicians (e.g. signs, symptoms, patients’ medical history and risks, and urine culture and dipstick results).

To ensure realization of its potential added value in improving appropriate antibiotic use, POCT should be carefully implemented in nursing home practice. However, any successful implementation of health care innovations is complex [18, 19]. Currently, POCT of inflammatory markers is not widely implemented in Dutch nursing homes. Some nursing homes are using CRP POCT in respiratory tract management, following the Dutch guideline on respiratory tract infections (RTIs) in primary care [20]. The only qualitative studies of POCT in primary care have so far involved general practitioners. Their opinions, expectations and experiences were mixed [21–23]. Perceived advantages included reduced diagnostic uncertainty, a more robust diagnosis and prognosis and a reduction of antibiotics prescriptions. Reported concerns related to the interpretation of the test, including the risk of false positives, false negatives and intermediate test results, and worries about becoming over-reliant on test results rather than on one’s professional expertise and clinical reasoning. Perceptions like these may considerably influence the implementation process and the appropriate use of POCT in practice. Hence, if POCT is sufficiently sensitive to support UTI diagnosis, and is to be implemented in nursing homes, it is crucial to explore the opinions, expectations and experiences of its potential users. So far, qualitative studies on the implementation of POCT in nursing homes are lacking. Therefore, the aim of this study was to explore the perceptions of care professionals in Dutch nursing homes regarding the role of POCT in UTI diagnosis and antibiotic treatment.

## Methods

### Design

In this qualitative study, we used semi-structured interviews to identify physicians’ and nurses’ perceptions of the proposed use of inflammatory marker POCT in the management of UTI in nursing home residents.

### Setting

The present study was conducted alongside the PROGRESS study (NL6293) [17], which aimed to assess the sensitivity of two inflammatory markers, CRP and PCT measured by POCT for diagnosing UTI. All three nursing home organizations that took part in the PROGESS study also agreed to participate in the present interview study. These organizations (A-C, referred to below as non-users) had not yet implemented inflammatory marker POCT in regular care. This is representative of the situation in most Dutch nursing homes. As experience with this type of testing was expected to influence the professionals’ perceptions, we also invited one nursing home organization that did not participate in the PROGRESS study, but had implemented inflammatory marker POCT for routine RTI management (organization D; referred to below as users).

### Sample

Within this convenience sample of nursing home organizations, we used purposive sampling to maximize the variety of respondents in the study [24]. This heterogeneity was intended to achieve an optimal understanding of the subject of our study. First, we selected 12 nursing homes from organizations A-C and one from organization D, in order to cover somatic, psychogeriatric and rehabilitation care. Next, we invited 18 care professionals, by e-mail or face-to-face, who worked at wards differing in size and nature, and who differed in years of work experience.

We selected both physicians (*n* = 12) and nurses (*n* = 6), as both professions were involved in UTI management at that time and expected to be involved in the future use of the inflammatory marker POCT [25]. In Dutch nursing homes, the final responsibility for POCT, diagnosing UTI and prescribing antibiotics rests with the resident physician or elderly care physician. These physicians can delegate tasks with respect to interpreting POCT to a nurse practitioner or a trained nurse. These nurses usually perform the test, monitor the nursing home resident, and inform the physician about the resident’s condition.

For an overview of the sample, see Table 1. At the time of the interviews, neither the researcher nor the respondents were aware of the results of the PROGRESS study.

**Table 1:** Characteristics of study respondents

Respondent	Profession	Years of work experience	Sex	Nursing home	Number of residents and type of ward	Number of sites	Experience with POCT
A-I	Nurse	25	Female	A-a	170 PG	2	No
A-II	Elderly care physician	4.5	Female	A-b	200 PG	2	No
A-III	Nurse	6	Female				No
A-IV	Elderly care physician	14	Female	A-c	130 PG	1	No
A-V	Elderly care physician in training	5	Male	A-d	80 RB; 30 PG; 30 SOM	1	No
A-VI	Elderly care physician	19	Female	A-f	25 RB	1	No
A-VII	Nurse	29	Female				No
A-VIII	Elderly care physician	18	Female	A-g	145 PG	2	No
B-I	Elderly care physician	35	Male				No
B-II	Elderly care physician in training	5	Female	^a^	115 RB; 290 PG; 120 SOM	5	No
B-III	Elderly care physician in training	0.5	Male				No
B-IV	Elderly care physician	16	Female				No
B-V	Nurse practitioner	24	Female				No
B-VI	Nurse	30	Female				No
C-I	Elderly care physician	14	Female	C-a	90 PG	1	No
C-II	Nurse	34	Female				No
D-I	Elderly care physician in training	1.5	Female	D-a	100 RB; 100 SOM	1	Yes
D-II	Elderly care physician	30	Male				Yes

*POCT* point-of-care testing, *PG* psychogeriatric, *RB* rehabilitation, *SOM* somatic

^a^ Physicians and nurses are not based at a single nursing home, but visit different nursing homes for consultations

### Data collection

In 2018 and 2019, semi-structured individual interviews were held by one of the researchers [SK] [26]. In addition to the skills in (medical) history taking acquired during her training as a physician, the researcher attended a five-day training course on qualitative research methods. As a result of her role in the PROGRESS study, the researcher knew most of the physicians and nurses in organizations A, B and C, but none of the professionals in organization D.

The interviews were held in the nursing homes where the professionals worked, and took 45 to 60 min each. No repeat interviews were performed. All but one of the interviews were audio-recorded, and during each of the interviews field notes were made. The researcher first explained the aim of the interview study, and that respondents would be de-identified in the report. She encouraged respondents to be honest and critical about the subject of the study. She then briefly introduced the aims and methods of inflammatory marker POCT. Next, she conducted the interview, using a brief guide that included both open questions and follow-up prompts so as to encourage further elaboration (see Additional file 1).

The interview guide was based on two theoretical sources. First, we used the Consolidated Framework for Implementation Research (CFIR) [27]. This framework distinguishes five domains of factors influencing the implementation of an innovation. Second, we applied Rogers’ Diffusion of Innovations Theory [28]. According to this theory, the diffusion of innovations is a stepwise process, including an adoption, implementation and consolidation stage. We used Rogers’ theory to select those factors from the CFIR that covered the first stages of the diffusion process, thereby reflecting the current stage of implementation of inflammatory marker POCT in Dutch nursing homes. This lead to the following themes for the interview guide: (1) the professionals’ understanding of the test, including its intended use (for whom, when and why?); (2) the perceived role and relative advantage of the test in UTI diagnosis, treatment and antimicrobial resistance; (3) the characteristics of the setting, including the needs of patients and their families, the work procedures and the organizational climate in the nursing home.

The interview guide was not formally pilot-tested. After the interviews, data on sex, profession and years of work experience were recorded. Participants did not check or correct the interview transcripts.

### Data storage

Data was collected pseudo-anonymously by using unique codes. The subject identification code list was stored securely. All source data (field notes, recordings and transcripts) was stored separately from coded data.

### Data analysis

The first two interviews were transcribed verbatim by the researcher immediately after the interview. This made her familiar with the data collected and helped her to reflect on and adjust her interviewing style. The other interviews were transcribed by an independent professional transcription company. Transcripts were analyzed after the data collection using the MaxQDA software package [29]. We used thematic content analysis [26].

The first phase of coding was directly based on the themes of the interview guide. Four transcripts were coded and discussed by two researchers [SK and JH] to ensure reliability. The coding tree (see Additional file 2) covered: (1) intended use of POCT (type of residents, symptoms, illness severity) and role of POCT (diagnosing, monitoring); (2) perceived POCT characteristics and advantages and disadvantages of POCT; (3) characteristics of healthcare workers in relation to POCT e.g. self-efficacy; (4) organizational aspects; and (5) implementation strategies.

In the second phase, the two researchers identified overarching themes. Four themes related to the respondents’ expectations about the added value of POCT in reducing diagnostic uncertainty in case of suspected UTI. These included the test’s application in case of non-specific urinary symptoms, its use as an objective measure for ‘ruling out’ UTI, its use as an objective measure for ‘ruling in’ UTI, and finding a balance between test results and clinical symptoms through clinical reasoning. A fifth theme concerned the use and added value of POCT in situations other than suspected UTI. These overarching themes guided the reporting of the study results, while we included considerations regarding the sensitivity and the specificity of POCT as a final, interwoven theme. The study results were based on the reports of all respondents, including physicians and nurses as well as non-users and users. Respondents were not invited to check or confirm these results.

## Results

### Respondents’ characteristics

All 18 physicians and nurses we invited agreed to participate (see Table 1). The sample included both non-users (*n* = 16, organizations A-C) and users (*n* = 2, organization D), and covered somatic, psychogeriatric as well as rehabilitation care. The majority of participants were female (14 out of 18). The median number of years of work experience was 17 (range 0.5–35 years).

### Role of POCT in urinary tract infections

#### Only added value in case of non-specific symptoms

About half of the respondents explicitly stated that, in nursing home residents presenting with specific urinary symptoms, inflammatory marker POCT (referred to below as POCT) has no added value as there is no diagnostic uncertainty, but that attaining such diagnostic certainty was only possible in a small minority of nursing home residents able to adequately express complaints.

*Well, if you’re not in doubt, then there’s no need, is there? If someone shows the full set [of symptoms], then in my view there’s no point in confirming it with POCT.* **A-IV**

*Then it’s fairly obvious. But the group who are able to express this clearly and accurately is very small.* **B-IV**

The other respondents implicitly agreed with this limitation of the scope of POCT, as they only discussed testing in the context of residents with non-specific symptoms.

#### Objective measure for ‘ruling out’ UTI

All respondents primarily expected POCT to decrease diagnostic uncertainty by ‘ruling out’ UTI in residents with non-specific symptoms. Negative test results were considered to be an objective measure of the absence of UTI.

*But sometimes you’re faced with people where you think: well, this is really very very different from what we usually see. And you then want to exclude [a UTI] some way or other, as it might still be that.* **A-II**

*So you want to have something more objective [...] regarding the severity of illness, to help you decide. So that [if the POCT] is below 20, you think, well, we can just as well wait and see.* **A-VIII**

But several respondents expressed concerns about false negative test results, which could lead to erroneously withholding antibiotic treatment in nursing home residents with a UTI.

*So I test it, and it’s a CRP [POCT] of 2. So at any rate you know it’s not an obvious infection. [But] I’m not sure it’s that reliable.* **A-V**

*The danger might be that someone is not treated who should be treated, as they would otherwise become more severely ill of their complaints persist.* **C-I**

Having POCT as an objective measure in addition to clinical reasoning was perceived as important to convince others, such as family members and professional caregivers, of the appropriateness of withholding antibiotic treatment from residents with non-specific symptoms.

*If POCT is zero, you can then say well, this is definitely not it. So you have a stronger argument. [ … ] You have an objective finding, in addition to what I see or hear myself. Not that I think that’s not reliable, but relatives often want a test or a scan.* **B-II**

However, some respondents also admitted that POCT as an objective measure was of additional importance to corroborate their own clinical reasoning, for instance because it could support them in taking another direction in their diagnostic reasoning than just considering UTI.

*You want to check that your clinical impression is right. We doctors like that. To have some proof.* **A-VI**

*And that enables you to change direction sooner with this, well, in this area.* **A-II**

Although POCT was perceived as helpful for ‘ruling out’ UTI in nursing home residents with non-specific symptoms, their symptoms generally persisted. Respondents reported that in such cases diagnostic uncertainty remained. Hence, this could induce a feeling of unease, which in turn could lead to prescribing antibiotics after all.

*But I would be [* … *] skeptical at first. Like, well, the CRP [POCT] is lower, so now I can’t treat them for UTI. [But] what then?* **C-I**

*And so you either start to treat because you feel you have to do something [ … ] Or you don’t, and then the complaints often persist anyway … And so at a later stage you still start to treat.* **A-V**

#### Objective measure for ‘ruling in’ UTI

Almost all respondents additionally thought POCT was a useful tool for ‘ruling in’ UTI. The inability of many nursing home residents to adequately express their symptoms made it difficult to rely on clinical reasoning alone. As an objective measure, POCT could reduce this diagnostic uncertainty, since positive test results would confirm UTI diagnosis and justify antibiotic treatment.

*That would be really helpful. [ … ] You can do an abdominal exam. But is it tender: is it constipation or a bladder infection? Or do they just dislike the fact that you’re touching their belly? So for us, diagnostics is still a kind of intuition, and such a test would actually …* **A-IV**

*And if I then also find an elevated CRP [POCT], that would be an argument to say this is really a UTI. As I don’t have any other instrument.* **B-I**

Some respondents also thought that POCT could speed up the diagnostic process, and thus the start of antibiotic treatment for UTI, as it is a ‘rapid test’, and its objective nature could compensate for suboptimal clinical attention.

*Perhaps it’s more a matter of seeing that if the POCT is elevated, you’ll give an antibiotic sooner, whereas that person would only become really ill tomorrow; so you [ … ] can get a timely start.* **D-I**

*But at [psychogeriatric wards] for instance, where there is less close clinical surveillance, there might be a group that you could treat a bit sooner.* **A-V**

Yet, in order to fulfil its supporting role in ‘ruling in’ UTI, it was acknowledged that POCT values should exceed a certain threshold in residents with a UTI, even in residents with non-severe illness or presenting with non-specific symptoms. The latter was doubted by some respondents.

*People may not be completely healthy, but not terribly ill either, so you think: it might be a UTI. But whether [ a POCT] would really rise that much in that kind of infection [ … ] I wonder.* **B-IV**

Despite the perceived advantage of POCT for ‘ruling in’ UTI, various respondents thought that positive test results might give rise to new diagnostic uncertainties. Due to the limited specificity of general inflammatory markers, positive results could also indicate the presence of other infections. Therefore, treatment decisions remained dependent on clinical reasoning.

*You don’t know where the infection is located. It doesn’t have a specific … It could be anywhere. [ … ] You get all kinds of infections with the same signs. [ … ] You have to look at that carefully. [ … ] So [POCT] is just an addition to the toolkit.* **B-I**

As a result, some respondents feared that the limited specificity of a POCT could lead to inadequate treatment, such as prescribing antibiotics for UTI while the resident is actually suffering from another infection.

*If you have someone with vague symptoms and a high [POCT], do you then know it’s a urinary tract infection? [ … ] The danger is that there’s an infection elsewhere, and that you treat them with nitrofurantoin and that that has no effect.* **B-IV**

Hence, in the case of non-specific symptoms, some respondents recommended either combining POCT with further examination of clinical symptoms to exclude infections other than UTI, or not to use POCT at all in residents suspected of having another infection besides UTI.

*You preferably want to get it in context as much as possible. So not someone who also happens to have a cough and has just arrived on the rehabilitation ward, and might have a cystitis. Cos then you don’t know what you’re measuring.* **A-V**

#### Balancing POCT and clinical reasoning

POCT was thought to reduce diagnostic uncertainty if the test results matched the respondents’ clinical reasoning. In case of disagreement between the two assessments, respondents reported different ways of balancing them against each other. Sometimes, clinical reasoning prevailed: respondents explained how they would either refrain from antibiotic treatment in residents who were not very ill despite positive tests results, or would start such treatment in severely ill residents despite negative test results.

*When I see a CRP of 100 and the patient doesn’t appear ill at all, I will not refer them to hospital straight away purely on [the basis of] this CRP.* **A-V**

*Sometimes you might do a POCT … And then you think, well, that’s not very high. And still the patient is so ill that you give them an antibiotic anyway.* **D-II**

In other situations of disagreement, POCT results would overrule clinical reasoning. For instance, some respondents reported they would start antibiotic treatment in response to positive test results in residents who were not very ill. An important reason to do so was the general frailty of the resident.

*And in case of doubt; if I do see an elevated CRP [POCT] that makes me think right, there’s something going on there. And especially if it’s a very vulnerable patient, then I’ll think: let’s start treatment anyway.* **B-II**

When balancing the results of POCT with the clinical work-up, some respondents acknowledged the importance of support for nursing home staff in the interpretation of POCT, given the characteristics of a particular test.

*I think it’s more important for us to get clinical training about how to interpret test results and when to use them. [ … ] How about the specificity and sensitivity, can you use it to rule out, can you use it to rule in?* **B-III**

*You ought to know the predictive value of a test. [ … ] It’s just that we tend to forget that all the time.* **D-II**

### Role of POCT in other situations

Several indications other than diagnosing UTI were mentioned for POCT. Firstly, respondents suggested that POCT could be useful to (1) demonstrate if a resident is suffering from any infection in general, as well as to (2) confirm specific infections that are common in nursing home residents.

*And also to decide whether someone is not the way they usually are, or we can’t find out exactly what’s the matter. Then it’s sometimes interesting to know, well, could [it] have to do with an infection?* **A-VIII**

*That would be good, as for instance cellulitis, that’s sometimes an unclear picture here at the nursing home. [ … ] Then you have a much more powerful argument to start antibiotics.* **A-V**

Secondly, respondents explained that POCT could be helpful in distinguishing infectious from non-infectious disorders with comparable clinical manifestations.

*Of course sometimes you get vague abdominal symptoms that make you think: is this a cholecystitis or gall stones or what? I could imagine that you might be able to differentiate with that too.* **A-IV**

Thirdly, POCT was regarded as a useful tool to indicate infection severity, to monitor the course of an infection, or to predict a resident’s prognosis in general. In such cases, POCT could, for instance, help to decide whether to consult a specialist or whether to refer a resident to an emergency department.

*Yeah, sometimes you get people who deteriorate, and you can’t put your finger on it. Is it an airways infection rather than a urinary infection? So you’re at a loss. And you then check which way it’s developing. For prognosis too.* **A-IV**

*If people are ill and you ask yourself can I safely leave this patient here or should I refer them to an orthopedist for a check-up as we’re thinking it might be an infection of the hip, for instance.* **D-II**

At the same time, most respondents questioned the evidence base for these other roles, and noted that cut-off values for interpreting POCT results should be, but were currently not, available for these additional applications.

*Except, well, it’s not really intended for, we don’t have official cut-off values. Well. So then it’s still your own interpretation of the overall …* **A-VIII**

*Well, so where’s the threshold? I think it’s very important to put it in context and link it to the evidence base.* **A-V**

## Discussion

### Summary

In our qualitative study, we examined the expected use and benefits of inflammatory marker POCT to support UTI diagnosis and appropriate antibiotic use in nursing home residents. The intended users expected that POCT would to some extent reduce diagnostic uncertainty in residents with non-specific symptoms. They primarily regarded POCT as an instrument to rule out UTI and withhold antibiotics accordingly. However, in the case of negative test results, they would still feel uncertain about how to deal with persistent non-specific symptoms. In addition, they expressed that using a sufficiently sensitive POCT could be helpful to rule in UTI and serve as additional confirmation to justify starting antibiotic treatment. However, in the case of positive test results, POCT was also expected to introduce new diagnostic uncertainties, due to the limited specificity of general inflammatory markers, which in the case of other infectious or inflammatory diseases could result in false positive findings. Because of the limited specificity, the intended users also perceived POCT as a useful tool to identify and monitor other infections that are common in nursing home residents. Irrespective of its purpose, respondents generally expected that POCT carried a risk of creating a false sense of confidence. Therefore, carefully balancing test results with clinical symptoms would remain important in UTI management in residents presenting with non-specific symptoms.

### Interpretation and implications

Physicians and nurses in our study primarily regarded POCT as a helpful tool to rule out UTI, which indicates that they are mainly in need of diagnostic evidence to withhold antibiotics in nursing home residents presenting with non-specific symptoms. Supportive diagnostics could corroborate their own decision-making, which currently relies solely on subjective assessments, and could strengthen their arguments towards care assistants and family members, who might demand antibiotics. In previous studies, general practitioners similarly emphasized that POCT for RTIs contributes to a more robust diagnosis and benefits the management of patients’ expectations about prescribing antibiotics [21–23, 30]. Although it has been argued that a highly sensitive test is enough to rule out UTI [31], the negative predictive value of a test in a particular setting should, formally speaking, be sufficient to distinguish true negative from false negative test results [32]. Negative and positive predictive values use the disease prevalence to determine the probability of the absence or presence of the disease. However, these predictive values hardly played a part in the accounts of the respondents in our study.

Our results indicate that if general inflammatory marker POCT is to be implemented in UTI management in nursing homes, attention needs to be paid to several issues. Firstly, it should be clear to the intended users how to interpret POCT results, given the positive and negative predictive values in the setting where the test is used. Secondly, health care professionals using POCT could benefit from additional communication training. Similar to studies in general practice [21–23], our respondents perceived POCT as an objective tool to persuade residents and their family members that withholding antibiotics is justified. However, our study also indicates that even after POCT is implemented, diagnostic and treatment uncertainty will remain an issue. Additional communication training is warranted, as care professionals were found to attach greater value to communication skills than to inflammatory marker POCT in ‘selling’ treatment decisions when faced with both options at the same time [33, 34]. Thirdly, even if negative POCT results could justifiably rule out UTI, our results showed that the symptoms often persist, so physicians remain uncertain about how to manage these patients. They may then start antibiotics after all, which counteracts the improved treatment decisions. More clarity is needed about alternative diagnostic and/or therapeutic strategies in these residents. In case such strategies are not available, attention is needed to learn how to accept persisting symptoms without prescribing antibiotics.

Apart from ruling *out*, respondents regarded POCT as a useful tool to rule *in* UTI. Yet, they noted that other infectious diseases that are prevalent in nursing homes could cause false positive test results due to the non-specific nature of inflammatory markers. In such cases, new diagnostic uncertainties could arise which again could misguide antibiotics treatment decisions. Compared to previous studies, this is a new finding, which applies especially to the unique population of nursing home residents with their limited ability to express specific symptoms compared to other (adult) populations [6, 7]. The question arises whether general inflammatory marker POCT in a nursing home setting could improve antibiotic prescribing without risking new improper antibiotic use. Given the preference of future users for both ruling in and ruling out UTI, it seems useful to focus on markers with suitable positive and negative predictive values in further test development. Certain urinary biomarkers, such as immune regulators (interleukin 6 and 8), polymorph-nuclear elastase, secretory IgA or bacterial virulence factors, which are currently under investigation, are potentially valuable to differentiate ASB from UTI [35, 36]. Still, based on our results, it is to be expected that diagnostic uncertainty will remain an issue at least to some extent, even with ideal diagnostic testing.

In view of the non-specific nature of inflammatory markers, various other potential uses of POCT were mentioned by our respondents, e.g., diagnosing and monitoring other infectious and inflammatory diseases. Although such an extension of the scope of POCT is in line with findings in general practice [21], several respondents in our study also expressed their concerns about using POCT in conditions where test performance is unknown. Indeed, without proven accuracy, POCT could create a false sense of confidence and carry the risk of unnecessary antibiotic treatment [37]. This finding again stresses that inflammatory marker POCT should be carefully implemented in order to improve antibiotic use in nursing homes. Apart from the indication for which POCT use is validated and thus appropriate, our findings indicate that implementation programs should also address the importance of clinical reasoning in addition to POCT results.

### Strengths and limitations

The number of respondents in our study was limited, and the analysis was not conducted in parallel to the data collection. Therefore, data saturation could not be ensured. Also, comparing different subgroups (i.e. non-users versus users; physicians versus nurses) was not possible. Such a comparison might be of interest in future studies. However, given our heterogeneous sample, we assume that our rich data adequately reflect the wide variety of perceptions regarding the added value and conditions for implementation of inflammatory marker POCT in the management of UTI in nursing home residents [26].

The sensitivity of POCT in UTI had not yet been established at the time of this study. It is unlikely that this has influenced our results, however, as most respondents mentioned sufficient sensitivity as a requirement for effective POCT use. Once adequately high sensitivity of POCT in UTI is established, further research in nursing homes should be conducted to gather additional information on interpreting cut-off levels and intermediate test results, as was done in previous studies in general practice [21, 23].

At the time of this study, POCT was not yet being used to diagnose UTI in nursing home residents. Only two of our respondents were actually working with an inflammatory marker POCT, although this was for suspected RTI. As gaining experience in using a new test may require some time, and perceptions on POCT may change during its use, further studies should explore perceptions during or after POCT implementation.

## Conclusions

Our qualitative study indicates that inflammatory marker POCT has limited perceived added value for UTI management in nursing home residents. Future users expected POCT to reduce existing diagnostic and treatment uncertainties to some extent, but also to introduce new such uncertainties, and therefore merely create a false sense of confidence. In order to improve UTI management, inflammatory marker POCT, if found sufficiently sensitive, should be implemented very carefully to ensure appropriate use. Developing UTI markers with sufficient negative and positive predictive values may be more beneficial in improving UTI management in nursing homes.

This manuscript was written in accordance with the COREQ reporting guidelines [38].

## Supplementary Information

**Additional file 1.** Interview guide

**Additional file 2.** Coding tree

## Data Availability

The datasets generated and/or analyzed during the current study are not publicly available, as this would carry the risk of breaching participant confidentiality. Access to the anonymized transcripts of the interview data is possible under restrictions. These include agreement between the study team and the requesting party on the purpose of the requested use, and the proposed methodologies for analyzing the obtained data. Requests for access should be submitted to the corresponding author. When granted, access will be under a CC-BY-NC-SA license.

## Abbreviations

ASB: Asymptomatic bacteriuria
CCMO: National Central Committee on Research involving Human Subjects CFIR Consolidated Framework for Implementation Research
CRP: C-reactive protein
GP: General practitioner
PCT: Procalcitonin
POCT: Point-of-Care testing RTI Respiratory Tract Infection
UTI: Urinary tract infection ZonMw The Netherlands Organization for Health Research and Development
