# Supplementary material for: “A false sense of confidence” The perceived role of inflammatory point-of-care testing in managing urinary tract infections in Dutch nursing homes: a qualitative study
Source: BMC Geriatr. 2020 Nov 4;20:450. doi: 10.1186/s12877-020-01853-9 (PMC7643302; doi:10.1186/s12877-020-01853-9)
Supplement: Supplementary file 1 — Additional file 1. Interview guide [file 12877_2020_1853_MOESM1_ESM.docx]

**Additional file 1 Interview guide**

- What do you think about using Point-Of-Care testing (POCT) to diagnose urinary tract infections?
- When would you perform POCT?
  - When would you perform POCT?
    - For which (suspected) diseases, signs or symptoms?
    - At what degree of disease suspicion would you perform POCT?
    - At what point in the diagnostic process?
  - For what clinical scenarios, or for which patients would you perform POCT?
    - What type of residents (psychogeriatric, somatic, rehabilitation)?
    - Residents that are able or unable to express their symptoms?
    - At what degree of disease severity would you perform POCT?
  - What is your primary aim in using POCT?
    - To decide on antibiotic use
    - To rule in or rule out disease, to monitor disease, or for other aims?
  - What would be the added value of POCT?
    - Usefulness
    - Supporting medical decisions
    - Reduction of antibiotics use
    - Communication towards other healthcare professionals
    - Communication towards patients or patients’ relatives
  - How would you interpret POCT results?
  - Which other healthcare professionals should be involved in the process of implementing POCT?

Are there other important aspects to consider prior to implementing POCT in your setting, i.e., the nursing home setting?
