# Supplementary material for: “A false sense of confidence” The perceived role of inflammatory point-of-care testing in managing urinary tract infections in Dutch nursing homes: a qualitative study
Source: BMC Geriatr. 2020 Nov 4;20:450. doi: 10.1186/s12877-020-01853-9 (PMC7643302; doi:10.1186/s12877-020-01853-9)
Supplement: Supplementary file 2 — Additional file 2. Coding tree [file 12877_2020_1853_MOESM2_ESM.docx]

**Additional file 2 Coding tree**

|  | **Description and sub-codes** |
| --- | --- |
| **Use of inflammatory marker POCT** | Urinary tract infections  Specific urinary symptoms  Non-specific symptoms  Systemic symptoms |
|  | Respiratory tract infections |
|  | Other infections |
|  | Role of testing  Diagnosis  Follow-up  Triage |
|  | Type of patients  Psychogeriatric  Somatic/rehabilitation |
|  | Users |
|  | Maintenance |
|  | Interpretation |
|  | Clinical reasoning |
|  | Moment of use in diagnostic pathway |
| **Intervention** | Perception of advantages  Reducing diagnostic uncertainty  Withholding antibiotic therapy  Justification for starting antibiotic therapy  Reducing time to start of antibiotic therapy  Convincing others |
|  | Risks  Improper use (type of disease)  Incompetent use  Unstandardized decision making Medicalizing nursing home setting |
|  | Evidence |
|  | Perceptions of diagnostic accuracy |
|  | Perceptions of validity |
|  | Complexity of POCT |
|  | Mobility of POC device |
|  | Vulnerability (error-prone) |
|  | Patient burden |
|  | Time to test result |
|  | Workload for users |
|  | Trialability (evaluation) |
|  | Visibility |
|  | Costs |
| **Individuals** | Familiarity with POCT |
|  | Skills (also expected) |
|  | Self-efficacy |
| **Organization** | Nursing home structure (locally) |
|  | Nursing home organization / regional health system |
|  | Culture |
|  | Guideline, evidence |
|  | Patients’ needs |
| **Implementation** | Future |
|  | Education |
|  | Quality assurance |

POCT: point-of-care testing
